# Floor and ceiling effects on the Montreal Cognitive Assessment in patients with Parkinson’s disease in Brazil

**DOI:** 10.1590/1980-5764-DN-2023-0022

**Published:** 2023-12-04

**Authors:** Brenda Hanae Bentes Koshimoto, Pedro Renato de Paula Brandão, Vanderci Borges, Henrique Ballalai Ferraz, Artur Francisco Schumacher-Schuh, Carlos Roberto de Mello Rieder, Maira Rozenfeld Olchik, Ignacio Fernandez Mata, Vitor Tumas, Bruno Lopes Santos-Lobato

**Affiliations:** 1 Universidade Federal do Pará, Instituto de Ciências Médicas, Belém PA, Brazil.; 2 Universidade de Brasília, Laboratório de Neurociências e Comportamento, Brasília DF, Brazil.; 3 Hospital Sírio-Libanês, Instituto de Ensino e Pesquisa, Brasília DF, Brazil.; 4 Universidade Federal de São Paulo, Departamento de Neurologia, São Paulo SP, Brazil.; 5 Universidade Federal do Rio Grande do Sul, Departamento de Farmacologia, Porto Alegre RS, Brazil.; 6 Hospital de Clínicas de Porto Alegre, Serviço de Neurologia, Porto Alegre RS, Brazil.; 7 Universidade Federal de Ciências da Saúde de Porto Alegre, Departamento de Neurologia, Porto Alegre RS, Brazil.; 8 Universidade Federal do Rio Grande do Sul, Departamento de Cirurgia e Ortopedia, Porto Alegre RS, Brazil.; 9 Lerner Research Institute, Genomic Medicine, Cleveland Clinic, Cleveland, OH, USA.; 10 Universidade de São Paulo, Faculdade de Medicina de Ribeirão Preto, Departamento de Neurociências e Ciências Comportamentais, Ribeirão Preto SP, Brazil.; 11 Hospital Ophir Loyola, Serviço de Neurologia, Belém PA, Brazil.

**Keywords:** Mental Status and Dementia Tests, Parkinson Disease, Data Accuracy, Brazil, Testes de Estado Mental e Demência, Doença de Parkinson, Confiabilidade dos Dados, Brasil

## Abstract

Parkinson’s disease (PD) is a common neurodegenerative disease associated with cognitive impairment. The Montreal Cognitive Assessment (MoCA) has been used as a recommended global cognition scale for patients with PD, but there are some concerns about its application, partially due to the floor and ceiling effects.

**Objective:**

To explore the floor and ceiling effects on the MoCA in patients with PD in Brazil.

**Methods:**

Cross-sectional study with data from patients with PD from five Brazilian Movement Disorders Clinics, excluding individuals with a possible diagnosis of dementia. We analyzed the total score of the MoCA, as well as its seven cognitive domains. The floor and ceiling effects were evaluated for the total MoCA score and domains. Multivariate analyses were performed to detect factors associated with floor and ceiling effects.

**Results:**

We evaluated data from 366 patients with PD and approximately 19% of individuals had less than five years of education. For the total MoCA score, there was no floor or ceiling effect. There was a floor effect in the abstraction and delayed memory recall domains in 20% of our sample. The ceiling effect was demonstrated in all domains (80.8% more common in naming and 89% orientation), except delayed recall. Education was the main factor associated with the floor and ceiling effects, independent of region, sex, age at evaluation, and disease duration.

**Conclusion:**

The floor and ceiling effects are present in specific domains of the MoCA in Brazil, with a strong impact on education. Further adaptations of the MoCA structure for underrepresented populations may reduce these negative effects.

## RESUMO

A doença de Parkinson (DP) é uma doença neurodegenerativa comum associada ao declínio cognitivo. A Avaliação Cognitiva de Montreal (*Montreal Cognitive Assessment* — MoCA) tem sido usada como uma escala de cognição global recomendada para pacientes com DP, mas existem algumas preocupações sobre sua aplicação, em parte pelos efeitos solo e teto.

**Objetivo:**

Explorar os efeitos solo e teto na MoCA em pacientes com DP no Brasil.

**Métodos:**

Estudo transversal com dados de pacientes com DP oriundos de cinco Clínicas de Distúrbios de Movimento no Brasil, excluindo-se pessoas com possível diagnóstico de demência. Nós analisamos a pontuação total da MoCA, assim como a de seus sete domínios cognitivos. Os efeitos solo e teto foram avaliados para a pontuação total da MoCA e seus domínios. Foram feitas análises multivariadas para a detecção de fatores associados os efeitos solo e teto.

**Resultados:**

Nós avaliamos dados de 366 pacientes com DP, e aproximadamente 19% das pessoas tinham menos que cinco anos de escolaridade. Para a pontuação total do MoCA, não houve efeito solo ou teto. Houve efeito solo nos domínios abstração e memória de evocação tardia em 20% de nossa amostra. O efeito teto foi demonstrado em todos os domínios (80,8% mais comum em nomeação e 89% orientação), com exceção de memória de evocação tardia. A educação foi o principal fator associado aos efeitos solo e teto, independentemente de região, sexo, idade na avaliação e duração da doença.

**Conclusão:**

Os efeitos solo e teto estão presentes em domínios específicos da MoCA no Brasil, com forte impacto da educação. Adaptações adicionais à estrutura da MoCA para populações vulneráveis podem reduzir esses efeitos negativos.

## INTRODUCTION

Parkinson’s disease (PD) is a neurodegenerative disease with rising incidence and prevalence worldwide and is the fastest-growing neurological condition^1^. PD is characterized by progressive motor and non-motor symptoms, causing disability, negative impacts on quality of life, and elevated costs for public health systems^2^. Cognitive impairment is a common issue in PD, with approximately 23–55% of patients having mild cognitive impairment (MCI)^3^, which is a risk factor for dementia^3^.

The Mini-Mental State Examination (MMSE) is the most widely used cognitive screening instrument for elderly people. Unfortunately, MMSE presents some limitations in detecting MCI, probably due to the lack of tasks involving a more complex memory test, executive functions, higher-level language abilities, and visual-spatial processing^4^. The Montreal Cognitive Assessment (MoCA) was developed to increase the sensitivity to detecting MCI, including cognitive domains less tested in the MMSE^4^. Afterward, the MoCA was proposed as a superior tool for detecting cognitive impairment than the MMSE when applied in patients with PD^5^. A memory test with more words and a longer delay to recall and inclusion of executive functions and visual-spatial tasks supposedly converted the MoCA into a cognitive screening better fitted for PD than the MMSE^5^. Currently, the MoCA is a recommended global cognition scale for PD by the International Parkinson and Movement Disorder Society^6^.

However, studies with low-educated populations showed that the MoCA requires literacy abilities and is strongly influenced by age and education^7–10^. The cut-off score for cognitive decline varies across its numerous official language versions^11^, and the 1-point addition to the score for individuals with less than 13 years of education may not be sufficient as a correction factor for different cultural and educational settings^12^.

Floor and ceiling effects are present in a test when a substantial proportion of individuals register the minimum or maximum score, respectively, impairing the test’s property to discriminate between subjects according to their true abilities^13^. Floor and ceiling effects may introduce bias in mean and variance estimates, which cause distortions in analytic methods.

The ceiling effect has been described for the MMSE, which impairs the discrimination of MCI in people with higher education and mild PD^5^. For the MoCA, the ceiling effect was not found in patients with PD^5^, but there is evidence of the floor effect in specific domains in these patients^14^. The present study aimed to explore the floor and ceiling effects on the MoCA in patients with PD in Brazil, a developing country with marked cultural diversity between regions. Furthermore, we evaluated if floor and ceiling effects were influenced by factors like education, regional diversity, sex, age, and disease duration.

## METHODS

### Study design and ethical approval

We conducted a multicentric, observational, and cross-sectional study with data from Brazilian patients with PD to evaluate the floor and ceiling effects on the MoCA. We included patients followed at four Movement Disorders Clinics in Brazil (Ribeirão Preto, São Paulo, Porto Alegre, and Belém), which are part of the Latin American Research Consortium on the Genetics of Parkinson’s Disease (LARGE-PD). Some patients from Ribeirão Preto were described in a previous study^14^. We also included patients from a fifth Movement Disorders Clinic (Brasília, Federal District). We enrolled patients between May 2007 and July 2022. The study was approved by the Ethics Committee from Hospital das Clínicas de Ribeirão Preto, and all participants provided written informed consent.

### Inclusion and exclusion criteria

All patients met the UK Parkinson’s Disease Society Brain Bank clinical diagnostic criteria for PD^15^ and were native Brazilian Portuguese speakers. To avoid including patients with a possible diagnosis of dementia, we excluded participants according to the MoCA cutoff scores recommended by the Scientific Department of Cognitive Neurology and Aging of the Brazilian Academy of Neurology, stratified by education level (no formal education: ≤8; 1–4 years: ≤15; 5–8 years: ≤16; 9–11 years: ≤19; ≥12 years: ≤21)^10,16^.

### The Montreal Cognitive Assessment

The MoCA is a brief assessment that measures seven cognitive domains: visual-spatial/executive functions (0–5 points), naming (0–3 points), attention (0–6 points), language (0–3 points), abstraction (0–2 points), delayed memory recall (0–5 points), and orientation (0–6 points)^4^. The total MoCA score ranges from 0 to 30, with lower scores indicating inferior cognitive performance. We added one point to the total score when individuals had less than 13 years of education. There is a validated version of the MoCA for Brazilian Portuguese^17^.

### Definitions of floor and ceiling effect

For the present study, we defined the floor effect when 15% or more of individuals from the sample achieved the score’s worst level and the ceiling effect when 15% or more of individuals had the best score^18^. The floor and ceiling effects were evaluated for the total MoCA score and their domains.

### Statistical analysis

To evaluate potential factors associated with floor and ceiling effects, we performed a multivariate logistic regression model and defined the presence of floor and ceiling as dependent variables. We selected site of origin, sex, age at evaluation, disease duration, and education (in years) as independent clinical variables. We used the Pearson’s chi-square test to compare categorical variables and the Kruskal-Wallis test for continuous variables. All analyses were performed using Statistical Package for Social Sciences (SPSS) for Windows, version 23.0 (SPSS Inc., Chicago, USA), and graphical representations were generated using the R software version 4.0.4 and the R package *ggplot2*.

## RESULTS

### Clinical characteristics of the sample

We recruited 573 patients with PD from five Brazilian centers and excluded 127 for possible dementia and 80 due to missing data. A total of 366 patients were eligible for analysis (Table 1). There was a high clinical heterogeneity between participants from each of the five centers according to sex, disease duration, and education level (Table 1). Approximately 80% of them had mild disease (Hoehn & Yahr stages 1 and 2), and less than 7% were in advanced stages (Hoehn & Yahr stages 4 and 5).

**Table 1:** Sample characterization and distribution of total score of the Montreal Cognitive Assessment of patients with Parkinson’s disease in five Brazilian centers.

Variable		Total (n=366)	Belém (n=90)	Ribeirão Preto (n=58)	São Paulo (n=74)	Porto Alegre (n=83)	Brasília (n=61)	p-value
Male sex, % (n)		59.3 (217)	62.2 (56)	58.6 (34)	67.6 (50)	36.1 (30)	77.0 (47)	**<0.001** *
Age at evaluation†		59 (52–67)	57 (50–71)	60 (50–71)	58 (52–66)	60 (52–67)	64 (52–71)	0.12‡
PD duration†		7 (4–11)	5 (3–10)	7 (3–13)	7 (5–11)	11 (6–14)	3 (2–6)	**<0.001** ‡
Hoehn & Yahr stage. % (n)	1	12 (44)	5.1 (5)	8.3 (5)	2.5 (2)	3.44 (3)	47.8 (29)	**<0.001** *
	2	67.4 (247)	78.2 (70)	77.8 (45)	71.6 (53)	56 (47)	52.1 (32)	
	3	13.6 (50)	9 (8)	8.3 (5)	23.5 (17)	24.5 (20)	0 (0)	
	4	5.73 (21)	7.1 (6)	5.6 (3)	1.2 (1)	12.9 (11)	0 (0)	
	5	1 (4)	0.6 (1)	0 (0)	1.2 (1)	3.01 (2)	0 (0)	
Years of education†		11 (6–13)	11 (8–11)	8 (4–13)	8 (4–11)	8 (5–12)	16 (11–18)	**<0.001** ‡
Education level, % (n)	No formal education	3 (11)	2.2 (2)	0 (0)	5.4 (4)	6 (5)	0 (0)	**<0.001** *
	1–4 years	16.7 (61)	7.8 (7)	32.8 (19)	31.1 (23)	12 (10)	3.3 (2)	
	5–8 years	22.4 (82)	20 (18)	22.4 (13)	23 (17)	37.3 (31)	4.9 (3)	
	9–11 years	27.3 (100)	54.4 (49)	17.2 (10)	20.3 (15)	19.3 (16)	16.4 (10)	
	≥12 years	30.6 (112)	15.6 (14)	27.6 (16)	20.3 (15)	25.3 (21)	75.4 (46)	
MoCA total score^b^	Median (IQR)	24 (21–26)	23 (21–26)	23 (21–26)	23 (20–26)	24 (21–26)	26 (24–27)	**<0.001** ‡
	Minimum-Maximum	11–30	13–30	18–30	13–30	11–30	20–30	

Notes:

Abbreviations: IQR, interquartile range; MoCA, Montreal Cognitive Assessment; PD, Parkinson’s disease.

* Pearson’s chi-square test comparing frequencies between patients from Brazilian centers

† Values in median (interquartile range)

‡ Kruskal-Wallis test comparing medians between patients from Brazilian centers. Bold numbers indicate p<0.05.

Participants had a median of 11 years of education (high school level). The frequency of individuals with less than five years of formal education was 19.7%, with 3% of illiterate individuals (no formal education). In Brasilia, the median number of years of education (16 years) was two times higher than that of the other three centers (8 years; Ribeirão Preto, São Paulo, and Porto Alegre), with only 3.3% of individuals with less than five years of education.

### Distribution of the total score and domains of the Montreal Cognitive Assessment between Brazilian centers

**Figure 1:**
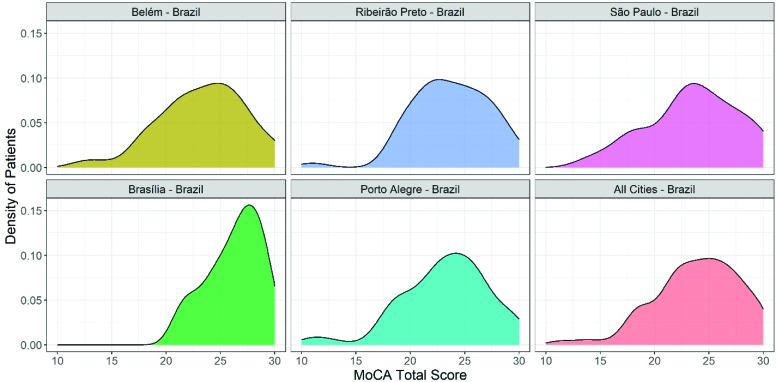
Distribution of the total score of the Montreal Cognitive Assessment in five Brazilian centers.

The distribution of the total score of the MoCA had a similar pattern in Belém, Ribeirão Preto, São Paulo, and Porto Alegre, with a peak density between 20–25 points (Table 1 and Figure 1). Brasília, the center with the highest frequency of individuals with education over 12 years, had a peak density close to 27 points. Regarding the domains of the MoCA, the scores varied significantly between centers and education levels (Table 2).

**Table 2:** Domains scores of the Montreal Cognitive Assessment in patients with Parkinson’s disease from five Brazilian centers, by education level.

Variable		Mean	SD	Mean	SD	Mean	SD	Mean	SD	Mean	SD	p-value*
Belém (n=90)		0y (n=2)		1–4y (n=7)		5–8y (n=18)		9–11y (n=49)		12+y (n=14)		
	Visual-spatial/executive functions	1.00	1.41	2.28	1.38	2.83	1.46	3.67	1.32	4.07	0.82	**0.001**
	Naming	1.33	1.15	2.28	0.75	2.44	0.70	2.77	0.42	2.85	0.53	**0.012**
	Attention	1.50	0.70	4.42	1.39	3.88	1.13	4.87	1.18	5.21	0.57	**<0.001**
	Language	0.00	0.00	1.71	0.75	1.44	0.98	2.12	0.90	2.14	0.94	**0.003**
	Abstraction	0.00	0.00	1.00	0.81	0.83	0.78	1.22	0.77	1.71	0.46	**0.003**
	Delayed recall	2.00	0.00	0.57	1.51	1.22	1.11	1.91	1.65	3.14	1.74	**0.003**
	Orientation	5.00	1.41	5.71	0.48	5.77	0.42	5.91	0.27	5.92	0.26	**0.007**
	MoCA Total Score	13.50	0.70	19.85	3.28	20.33	2.78	24.40	3.07	25.92	2.49	**<0.001**
Ribeirão Preto (n=58)		0y (n=0)		1–4y (n=19)		5–8y (n=13)		9–11y (n=10)		12+y (n=16)		
	Visual-spatial/executive functions	NA	NA	2.84	1.46	2.69	1.37	3.20	1.03	4.12	1.02	**0.012**
	Naming	NA	NA	2.89	0.31	2.46	0.66	2.90	0.31	2.87	0.34	**0.023**
	Attention	NA	NA	4.31	1.52	4.76	1.09	4.80	0.91	5.37	0.80	0.080
	Language	NA	NA	1.68	0.82	1.69	0.94	1.70	1.15	2.37	0.61	0.070
	Abstraction	NA	NA	0.84	0.76	0.76	0.92	1.20	0.91	1.75	0.44	**0.002**
	Delayed recall	NA	NA	2.42	1.38	2.15	1.51	2.90	1.19	3.12	1.36	0.230
	Orientation	NA	NA	5.84	0.50	5.92	0.27	5.46	1.24	5.95	0.21	0.440
	MoCA Total Score	NA	NA	21.84	3.18	21.46	3.35	23.70	2.49	25.68	2.21	**<0.001**
São Paulo (n=74)		0y (n=4)		1–4y (n=23)		5–8y (n=17)		9–11y (n=15)		12+y (n=15)		
	Visual-spatial/executive functions	1.25	0.95	2.08	1.47	2.94	1.19	3.93	1.16	4.26	1.22	**<0.001**
	Naming	2.75	0.50	2.82	0.38	2.82	0.39	2.86	0.35	3.00	0.00	0.520
	Attention	2.75	1.50	3.95	1.49	4.88	0.85	5.40	0.63	5.53	0.83	**<0.001**
	Language	0.75	0.50	1.82	0.75	2.17	1.07	1.93	1.03	2.40	0.94	**0.014**
	Abstraction	0.50	1.00	0.65	0.64	1.17	0.88	1.80	0.41	1.73	0.70	**<0.001**
	Delayed recall	0.00	0.00	1.82	1.61	2.11	1.57	1.93	1.65	3.06	1.33	**0.005**
	Orientation	4.75	2.50	5.82	0.38	5.88	0.33	5.86	0.51	6.00	0.00	**0.017**
	MoCA Total Score	13.75	0.95	20.00	2.74	23.00	2.78	24.73	3.03	26.13	2.64	**<0.001**
Porto Alegre (n=83)		0y (n=5)		1–4y (n=10)		5–8y (n=31)		9–11y (n=16)		12+y (n=21)		
	Visual-spatial/executive functions	1.80	2.04	1.70	1.88	2.61	1.66	3.12	1.58	3.76	1.22	**0.007**
	Naming	2.60	0.64	2.80	0.42	2.70	0.58	2.83	0.46	2.90	0.30	0.160
	Attention	2.80	1.50	3.80	1.22	4.67	1.01	4.87	1.25	4.61	1.24	**0.006**
	Language	1.40	0.54	1.80	0.42	2.09	0.65	2.31	0.70	2.33	0.73	**0.023**
	Abstraction	0.20	0.44	1.00	0.47	1.22	0.71	1.68	0.47	1.47	0.67	**<0.001**
	Delayed recall	0.60	0.54	2.10	1.85	1.61	1.58	2.68	1.16	3.14	1.52	**<0.001**
	Orientation	5.00	1.41	5.80	0.42	5.83	0.45	5.87	0.51	5.90	0.30	**0.010**
	MoCA Total Score	16.00	6.08	20.90	2.46	22.70	3.44	25.50	3.07	25.66	3.05	**<0.001**
Brasília (n=61)		0y (n = 0)		1–4y (n = 2)		5–8y (n = 3)		9–11y (n = 10)		12+y (n = 16)		
	Visual-spatial/executive functions	NA	NA	4.00	1.41	3.66	2.30	3.20	1.39	4.36	0.99	**0.037**
	Naming	NA	NA	2.00	0.00	2.66	0.57	2.60	0.51	2.93	0.24	**<0.001**
	Attention	NA	NA	5.00	0.00	4.33	0.98	5.10	0.87	5.32	0.94	0.330
	Language	NA	NA	2.50	0.70	2.00	1.00	1.70	0.94	2.19	0.80	0.340
	Abstraction	NA	NA	1.50	0.70	1.33	0.57	1.40	0.69	1.78	0.51	0.140
	Delayed recall	NA	NA	1.50	2.12	3.00	1.73	2.70	1.94	3.30	1.22	0.230
	Orientation	NA	NA	5.50	0.70	6.00	0.00	5.90	0.31	5.95	0.20	**0.070**
	MoCA Total Score	NA	NA	25.00	4.24	24.00	4.00	23.60	2.91	26.10	2.14	**0.008**

Notes:

Abbreviations: MoCA, Montreal Cognitive Assessment; NA, not available; SD, standard deviation.

* Analysis of variance comparing domain scores between education levels: 0y, 1–4y, 5–8y, 9–11y, and 12+y indicate the education level in years. Bold numbers indicate p<0.05.

### Floor and ceiling effect for the total score and domains of the Montreal Cognitive Assessment in patients with Parkinson’s disease in Brazilian centers

**Figure 2:**
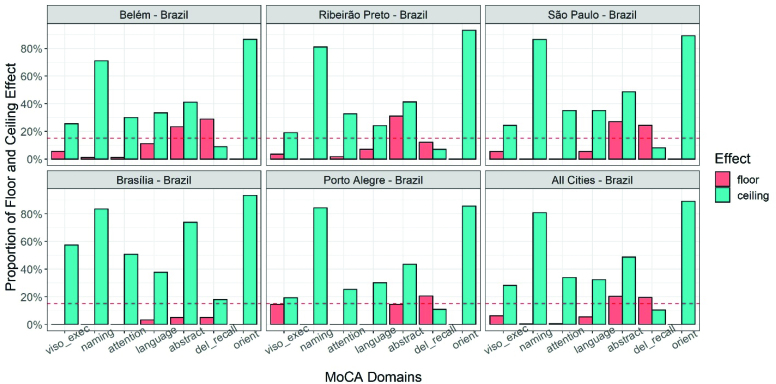
Proportion of floor and ceiling effects on the Montreal Cognitive Assessment domains in five Brazilian centers.

For the total MoCA score, there was no ceiling effect (only 21 patients scored 30/30; 3.27%) in our sample, neither was detected floor effect due to the exclusion of patients with possible dementia. According to the domains of the MoCA, there was an overall floor effect in abstraction (20.2%) and delayed memory recall (19.3%) and an overall ceiling effect for all domains except delayed recall (10.3%), which was more common in naming (80.8%) and orientation (89%) (Figure 2).

Red bars over the red dotted line indicate the cutoff value for floor and ceiling effects.

Among centers, the floor effect in abstraction was detected in three of them (Belém 23.3%; Ribeirão Preto 31%; São Paulo 27%), as well as in delayed recall (Belém 28.8%; São Paulo 24.3%; Porto Alegre 20.4%). Brasilia was the unique center with a ceiling effect in all domains (Figure 2), probably due to the participants’ high level of education.

### Factors associated with floor and ceiling effect for domains of the Montreal Cognitive Assessment in patients with Parkinson’s disease

After adjustment for the site of origin, sex, age at evaluation, and disease duration, the multivariate analysis showed that education was inversely associated with the floor effect for visual-spatial/executive functions, language, abstraction, delayed memory recall, and orientation (Table 3). Also, education was directly associated with the ceiling effect for all domains after adjustment, and female sex was associated with the floor effect for abstraction and with the ceiling effect for orientation (Table 3).

**Table 3:** Associations between clinical variables and presence of floor and ceiling effect on domains of the Montreal Cognitive Assessment in patients with Parkinson’s disease.

Domains/Clinical variables		Floor effect				Ceiling effect			
		B	aOR	95%CI	p-value	B	aOR	95%CI	p-value
Visual-spatial/executive functions	Female sex	0.223	1.25	0.47–3.29	0.65	0.149	1.16	0.62–2.14	0.63
	Age at evaluation	-0.006	0.99	0.94–1.04	0.82	-0.007	0.99	0.96–1.02	0.63
	Disease duration	0.05	1.05	0.97–1.14	0.16	-0.009	0.99	0.93–1.05	0.75
	Years of education	-0.222	0.80	0.70–0.91	**0.001**	0.190	1.20	1.12–1.29	<0.0001
Naming	Female sex	37.8	0.00	0.00–0.00	0.99	-0.266	0.76	0.41–1.42	0.39
	Age at evaluation	-0.954	0.38	0.00–0.00	0.98	-0.007	0.99	0.96–1.02	0.65
	Disease duration	-12.54	0.00	0.00–0.00	0.97	-0.036	0.96	0.91–1.01	0.20
	Years of education	-6.249	0.00	0.00–0.00	0.97	0.147	1.15	1.07–1.25	<0.0001
Attention	Female sex	33.823	0.00	0.00–0.00	0.84	-0.031	0.97	0.56–1.65	0.91
	Age at evaluation	-0.331	0.71	0.31–1.66	0.43	-0.001	0.99	0.97–1.02	0.96
	Disease duration	0.766	2.15	0.81–5.69	0.12	0.005	1.00	0.95–1.05	0.83
	Years of education	-1.933	0.14	0.01–2.60	0.19	0.126	1.13	1.07–1.20	<0.0001
Language	Female sex	0.059	1.06	0.37–2.99	0.91	-0.053	0.94	0.55–1.61	0.84
	Age at evaluation	-0.021	0.97	0.93–1.02	0.38	-0.017	0.98	0.95–1.00	0.17
	Disease duration	-0.003	0.99	0.91–1.09	0.95	-0.018	0.98	0.93–1.03	0.48
	Years of education	-0.151	0.86	0.75–0.97	**0.022**	0.108	1.11	1.05–1.17	<0.0001
Abstraction	Female sex	-0.862	0.42	0.21–0.81	**0.01**	0.355	1.42	0.82–2.45	0.20
	Age at evaluation	0.022	1.02	0.99–1.05	0.15	-0.013	0.98	0.96–1.01	0.31
	Disease duration	0.036	1.03	0.98–1.09	0.19	-0.023	0.97	0.92–1.02	0.38
	Years of education	-0.25	0.77	0.71–0.84	**<0.0001**	0.241	1.27	1.18–1.36	<0.0001
Delayed memory recall	Female sex	-0.342	0.71	0.38–1.31	0.27	0.187	1.20	0.52–2.78	0.72
	Age at evaluation	-0.008	0.99	0.96–1.02	0.61	0.007	1.00	0.96–1.04	0.66
	Disease duration	0.019	1.01	0.96–1.07	0.50	-0.007	0.99	0.91–1.07	0.87
	Years of education	-0.198	0.82	0.75–0.88	**<0.0001**	0.111	1.11	1.02–1.21	0.008
Orientation	Female sex	-0.342	0.71	0.38–1.31	0.27	-0.814	0.44	0.20–0.95	0.03
	Age at evaluation	-0.008	0.99	0.96–1.02	0.61	-0.017	0.98	0.94–1.02	0.37
	Disease duration	0.019	1.01	0.96–1.07	0.50	-0.048	0.95	0.89–1.01	0.15
	Years of education	-0.198	0.82	0.75–0.88	**<0.0001**	0.132	1.14	1.04–1.25	0.005

Note:

Abbreviations: B, regression coefficient; aOR, adjusted odds ratio; CI, confidence interval.

The results for “site of origin” were not shown in this table but the variable was included in the multivariate analyses. Bold numbers indicate p<0.05.

## DISCUSSION

This multicentric study with patients with PD without dementia in Brazil showed that the floor effect in the MoCA was present in abstraction and delayed memory recall domains in approximately 20% of our sample. The ceiling effect was demonstrated in almost all evaluated domains, with the exception of delayed recall. These effects were not detected in the total score of the MoCA. Education was the main factor associated with the floor and ceiling effect in most domains, independent of region, sex, age at evaluation, and disease duration. Only one-fifth of individuals had less than five years of formal education, including illiteracy.

As described previously, our results showed a higher prevalence of the ceiling than floor effect in specific domains of the MoCA score^18^. The floor effect in abstraction and delayed memory recall domains was already described in Southeastern Brazil, but also in the subtopics of attention and language^14^. The authors suggest that emphasizing the instructions for patients and including adaptations for our population could reduce the number of individuals scoring zero. The same pattern was reported for the ceiling effect affecting all domains, except delayed memory.

Floor/ceiling effects on cognitive tests may cause adverse impacts because the measurement of part of the sample’s true cognition (ability) is represented by censored data (observations partially known). Censoring reduces the capacity to highlight differences among lower- and higher-scoring individuals, concealing the proper range and variability of the measured cognitive function. Therefore, the exact levels of cognition are unknown due to these detrimental effects. Furthermore, floor/ceiling effects lead to a non-normal distribution of scores, artificial means and standard deviations, and weaken the reliability and validity of the analysis. Most used statistical tests are not adjusted for the censoring caused by floor/ceiling effects^13,19^.

Classically, floor/ceiling effects are described in total test scores, not in domains/subscales. As a neuropsychological battery, the MoCA may also be interpreted according to its specific domains. Compared to the MMSE, the MoCA was conceived to increase the detection of mild cognitive impairment, mainly based on visual-spatial/executive functions and delayed memory recall. Thus, it is reasonable to explore the presence of floor/ceiling effects in each domain.

Delayed memory recall is a crucial domain for neuropsychological batteries commonly used to diagnose cognitive decline in clinical practice. In the development of the MoCA, the authors proposed a memory test with more words, fewer learning trials, and a longer delay before recall than the MMSE^4^. According to our data, these adaptations may explain the floor effect in this domain.

In a sample of elderly individuals without PD from Tremembé, Brazil, with 68% of individuals having less than five years of education, the mean delayed memory recall subscores were lower than those seen in our results (no education: 0.8; 1–4 years: 0.94; 5–8 years: 1.79; 9–11 years: 1.6; ≥12 years: 2.13)^10^. In the Colombian city of Manizales, the authors also reported low mean delayed memory recall subscores in a sample with 49% of aged individuals without PD with less than five years of education (<5 years of education: 1.2; 5 years: 0.89; >5 years: 1.53)^7^.

Aiming to enhance frontal lobe-related tasks in the MoCA, the abstraction domain is based on similarity questions. Our low mean abstraction subscores were similar to data from Tremembé, Brazil, and Manizales, Colombia (Brazil — no education: 0.2; 1–4 years: 0.57; 5–8 years: 1.02; 9–11 years: 1.18; ≥12 years: 1.46; Colombia — <5 years of education: 0.57; 5 years: 0.87; >5 years: 1.3)^7,10^.

This is the first study where the MoCA and its domains were evaluated in patients with PD from four Brazilian regions (North, Midwest, Southeast, and South). Brazil’s socioeconomic, cultural, and linguistic diversity is represented in our sample, exploring the impact that cultural background may have on cognitive tests. Except for Brasilia, the regional diversity of included centers had a low impact on the MoCA total score and domains. We consider that the specific pattern of high scores and ceiling effects in all MoCA domains in Brasilia is probably due to the higher education level of its participants. Multivariate analyses showed no association between region heterogeneity and the presence of floor and ceiling effects.

Our sample had a low proportion of participants in the intermediate and advanced stages of disease (Hoehn & Yahr stages 3 to 5; 20.4%). Therefore, the impact of motor impairments on visual-spatial/executive functions domain-associated tasks (such as the trail-making test, copy of the cube, and clock drawing test) was probably limited.

Regarding the impact of education on the MoCA score, a recent study reinforced that high accuracy in discriminating normal individuals from people with dementia depends on the use of specific cutoff values stratified by education level^10^. For domains, our data from multivariate analyses showed a strong association of floor and ceiling effects with years of education, regardless of the socioeconomic and cultural diversity of Brazilian regions. In the MMSE, education is associated with floor/ceiling effects. The floor effect is frequent in people with low education, and the ceiling effect occurs mainly in highly educated individuals^20^.

Considering that approximately 16% of the global population may be illiterate and the low levels of education in developing countries, such as Brazil, the MoCA-Basic was designed to be applied to elderly adults in low-education settings, eliminating literacy-dependent tasks and adapting other tasks less dependent on education^21^. The MoCA-Basic scores did not differ according to educational level, without association with education or age^21^. There is no validation or normative data for the Brazilian Portuguese MoCA-Basic.

As a limitation, the absence of a formal cognitive status for all Brazilian centers did not assure the exclusion of all patients with dementia. Further, including individuals without PD would help understand the role of floor/ceiling effects in people with PD and controls. Also, we had a small sample of people with less than five years of education compared to previous works in underrepresented regions^7,10^, which could have affected our analyses. The low number of participants with less than five years of education was also reported in other studies with MoCA in PD^22,23^, which may be related to a selection bias favoring high-educated people. The lack of a control group without PD and the low proportion of people with less than five years of education impair the generalizability of our findings on the floor and ceiling effects to a larger population. The strengths of our study were the large sample size and the socioeconomic and cultural diversity of individuals from different Brazilian regions.

In conclusion, the present study showed the presence of floor and ceiling effects in all domains of the MoCA, with a strong impact on education. Further adaptations of the MoCA structure, based on statistical methods such as item response theory, may reduce the floor/ceiling effects.
